# Association between socioeconomic positions and overweight/obesity in rural Nepal

**DOI:** 10.3389/fnut.2022.952665

**Published:** 2022-09-09

**Authors:** Sanju Bhattarai, Rikke Nerhus Larsen, Archana Shrestha, Biraj Karmacharya, Abhijit Sen

**Affiliations:** ^1^Department of Public Health and Nursing, Norwegian University of Science and Technology, Trondheim, Norway; ^2^Institute of Implementation Science and Health, Kathmandu, Nepal; ^3^Department of Public Health, Kathmandu University School of Medical Sciences, Dhulikhel, Nepal; ^4^Department of Chronic Disease Epidemiology, Center of Methods for Implementation and Prevention Science, Yale School of Public Health, New Haven, CT, United States; ^5^Oral Health Services and Research Center, TkMidt, Trondheim, Norway

**Keywords:** obesity, epidemiology - descriptive, rural, Nepal, socioeconomic

## Abstract

**Introduction:**

Obesity and its association with socioeconomic factors are well-established. However, the gradient of this relationship among rural populations in low- and middle-income countries such as Nepal is not fully understood. We sought to assess the association of socioeconomic factors (education, income, and employment status) with overweight/obesity.

**Methods:**

This cross-sectional study analyzed data from 260 participants aged ≥18 years and attending a rural health center in Dolakha, Nepal. Self-reported data on demographic, socioeconomic, and lifestyle factors was collected, and weight and height were measured for all the study participants. Those with a body mass index of <25 kg/m2 were regarded as non-overweight/obese and those with ≥25 kg/m2 were regarded as overweight/obese. Poisson regression models were used to estimate prevalence ratios and corresponding 95% confidence intervals to assess the association between socioeconomic factors and overweight/obesity. In addition, we assessed the effect of modification by age and gender to study the effect of socioeconomic factors on overweight/obesity.

**Results:**

The age-standardized prevalence of overweight/obesity was higher for individuals with higher education (23%) and high-income (32%) and those who were unemployed (42%). Compared to the low-income and no formal education groups, the prevalence ratio of overweight/ obesity was 1.69 and 2.27 times more for those belonging to the high-income and high school and above groups, respectively. No evidence of effect modification by gender and age was observed.

**Conclusions:**

Socioeconomic factors, education, and income were positively associated with overweight/obesity prevalence in rural Nepal. Further large studies using longitudinal settings are necessary to replicate our findings.

## Introduction

Obesity, a common risk factor for major non-communicable diseases (NCDs) has tripled in the last 4 decades (1–3). In 2016, more than 1.9 billion adults were overweight worldwide (4), and the number is still increasing. In Nepal, the prevalence of overweight and obesity (OWOB) also increased from 21% in 2013 to 29% in 2019 (5, 6). Therefore, understanding the burden of OWOB is important to develop effective strategies to halt obesity-associated several adverse health outcomes.

In low-income countries like Nepal, socioeconomic development drives food choices and diet pattern (7), and as a country progresses, obesity burden shifts from high to low income groups (8–10). Gradual economic prosperity has triggered nutrition transition shifting dietary patterns from home-produced food to easily available processed food contributing to the burden of OBOW and NCDs in Nepal (11).

Understanding the role of socioeconomic status (SES) in explaining food behavior that determines an individual's body weight is important (7). Individuals with low SES status is associated with increased risk of obesity in high-income nations (12, 13) while in LMICs (14, 15) results are mixed. For instance, findings from cross-sectional studies suggested that educated and affluent Nepalese (16), South Asian women (17), and Indians (18) were more likely to be overweight or obese. Likewise, being employed was positively associated with OWOB in Nepal (19), Mexico (20), and South India (21). On the other hand, obesity was reported to be inversely associated with education level and income in Argentina (22) and Iran (23). Furthermore, a national survey conducted in Nepal suggested that the prevalence of OWOB was more among affluent individuals living in urban hills (5), while a survey conducted among 341 Nepalese bureaucrats reported 33.4% of the participants to be either overweight or obese (24). The possible reason of higher OWOB prevalence in urban Nepal could be high consumption of energy-dense and cheap fatty foods as well as being physically less active (25, 26), whereas the lower prevalence of obesity seen among rural individuals might be due to engagement in physically demanding jobs (27).

Nepal has experienced considerable economic growth in recent years, and in 2015, the average income was $2,500 GDP per capita (gross domestic product per capita) (28). The impact of economic growth on obesity in different SES groups remains unclear. Compared to the rural women of Sherpa ethnicity, urban women had higher body mass index (BMI) (29), and this difference is the result of increase in income and less energy expenditure in the urban population (26). Therefore, a study to assess the association between SES and OWOB in rural Nepal, where 80% of the Nepalese population reside, is necessary. We recently published an article reporting a positive association between SES and hypertension (30). In this study, we used data from the same study (30) on individuals visiting a primary health center in rural Nepal to assess the association between SES and two other highly prevalent comorbid conditions, i.e., overweight and obesity.

## Methods

The detail of the study design and the methodology used for this study are published elsewhere (30).

### Study setting

The study was conducted in Kirnetar health center in Dolakha district in Nepal, providing primary health services to eight rural villages in its proximity. It was an opportunistic screening. The health center, established in 2012, provides primary-level health services 6 days a week including 24-h emergency services.

### Study design and population

A cross-sectional study was conducted among 260 individuals who visited the health center for clinical examination or to purchase medicine from October to December 2016. Participants over 18 years were included in the study, but those who were pregnant were excluded.

### Data collection

All the recruited participants were interviewed by trained enumerators. Self-reported data on demographic and socioeconomic factors, clinical history, lifestyle, and dietary factors were collected using a validated STEPS questionnaire (5). The participants were asked to stand (without footwear, jackets, and sweaters) on an instrument placed on a flat floor to measure weight (in kg) using BOSCH Electronic Scale PPWA4201. Similarly, the participants were asked to stand tall with heels and head against the measuring tape placed on the wall (without footwear, cap and hat) and the lineal measurement on the top point of the head was measured to the nearest 0.05 cm (5).

### Outcome

BMI was computed by dividing the weight (in kg) by the squared value of height (in m) and categorized as underweight or normal weight (<25 kg/m^2^), overweight (25–29.9 kg/m^2^), or obese (≥30 kg/m^2^) according to WHO recommendation. For analyses, we collapsed BMI categories into two groups, i.e., non-obese (BMI <25 kg/m^2^) and overweight and obese (BMI ≥25 kg/m^2^).

### Exposures

#### Income

Per capita annual income was calculated by asking the total combined household income (in Nepalese rupee) in the year preceding the survey and dividing it by the total number of household members. Annual income was categorized into tertiles (low: 0–6,000, middle: 6,250–32,571, and high: 33,333–625,000 Nepalese Rupees).

#### Education

Participants who reported that they did not attend school were confined to the “*no formal education*” group; those who had at least 1 year of formal school including those who did not complete high school were confined to the “*less than high school*” group, and those who had completed high school or beyond were confined to the “*high school and above*” group.

Employment status: this variable was classified into three groups: farming (agricultural task), employed (government/non-government employees, self-employed people), or unemployed (retired, students, unpaid, unable to work, unemployed, homemakers).

### Covariates

Sociodemographic variables include age (in years), gender (males, females), marital status (yes, no), and ethnicity (Dalit, Brahmin, Chettri, others). Lifestyle-related variables include both smoke or smokeless tobacco use (never-users, current, former users); alcohol intake (drinking <1 glass per week, 1–3 glasses/week, >3 standard drinks/week were categorized as “low drinkers,” “moderate drinkers,” or “heavy drinkers,” respectively). Physical activity was assessed using Global Physical Activity Questionnaire (31) (≥ 600 metabolic equivalent minutes (MET) and < 600 MET were categorized as adequate and inadequate, respectively), as well as fruits and vegetables servings (<2, 2–4, and >4 servings per day).

### Statistical analysis

The descriptive data were presented as frequencies and percentages for categorical variables and mean and SD for continuous variables. To assess the association between socioeconomic positions and prevalence of OWOB, we used modified Poisson regression models with robust standard errors (32) to estimate prevalence ratio (PR) with corresponding 95% CI. We fitted the Poisson regression models to estimate PR because odds ratio provides an overestimated approximation of the risk when the prevalence of outcome of interest is common (≥10%) (33). Two models were constructed. Model 1 was unadjusted, and model 2 was adjusted for age (in years), gender (male, female), marital status (married, unmarried), and ethnicity (Brahmin, Chettri, Dalits, Other). The analyses of the association between SES and OWOB was stratified by gender (male vs. female) and age (<50 vs. ≥50 years). The statistical interaction was assessed by likelihood ratio test incorporating product terms of (1) categories of SES × age and (2) categories of SES × gender in the model. All the statistical analyses were performed using Stata/IC 14 (Stata Corp, College Station, TX, United States).

## Results

The sociodemographic and lifestyle characteristics of the 260 participants are presented in Table 1. The mean age of the study population was 45 years, 48.5% were women and 24.5% were OWOB. The prevalence of OWOB were higher among males, Dalit ethnicity, married, high level of education, high income and employed. Furthermore, the prevalence of OWOB were higher among those who consumed <2 servings of fruits and vegetables per day and those who were non-tobacco users, moderate drinkers, and less physically active.

**Table 1:** Distribution of sociodemographic, lifestyle, and SES factors by obesity status.

	**Total *N* = 260**	**Non-obese *N* = 196**	**Overweight and obese *N* = 64**
**Gender**	N	N (%)	N (%)
Male	134	93 (73.81)	33 (26.19)
Female	126	103 (76.87)	31(23.13)
**Age groups (categories)**			
18–34 years	77	58 (75.32)	19 (24.68)
35–49 years	88	60 (68.18)	28 (31.82)
50–65 years	55	47 (85.45)	8 (14.55)
66 years and above	40	31 (77.50)	9 (22.50)
Age in years, Mean (±SD)	45 (±16.42)	45.83 (±16.98)	44.44 (±14.60)
**Marital status**			
Unmarried	38	34 (89.47)	4 (10.53)
Married	222	162 (72.97)	60 (27.03)
**Ethnicity**			
Brahmin Chettri	173	133 (76.88)	40 (23.12)
Dalits	35	25 (71.43)	10 (28.57)
Others	52	38 (73.08)	14 (26.92)
**Education**			
No formal education	113	92(81.42)	21 (18.58)
Less than high school	106	80 (75.47)	26 (24.53)
High school or more	41	24 (58.54)	17 (41.46)
**Income**			
Low income	87	73 (83.91)	14 (16.09)
Middle income	87	68 (78.16)	19 (21.84)
High income	86	55 (63.95)	31 (36.05)
**Annual income median (IQR), NRS**	16,733 (35,994)	15,833 (32,666)	30,000 (47,428)
**Employment status**			
Unemployed	59	42 (71.19)	17 (28.81)
Farming	128	108 (84.38)	20 (15.63)
Employed	73	46 (63.01)	27 (36.99)
***Lifestyle factors***			
**Tobacco use**			
Never	108	72 (66.67)	36 (33.33)
Current	60	52 (86.67)	8 (13.33)
Former	92	72 (78.26)	20 (21.74)
**Alcohol intake**			
Never	195	152 (77.95)	43 (22.05)
Low (<1 glass per week)	12	9 (75.00)	3 (25.00)
Moderate (1–3 glass per week)	14	9 (64.29)	5 (35.71)
High (>3 glass per week)	39	26(66.67)	13 (33.33)
**Physical activity**			
MET^*^ <600 min/week	26	18 (69.23)	8 (30.77)
MET ≥600 min/week	234	178 (76.07)	56 (23.93)
**Fruits and vegetables servings**			
<2 servings per day	35	22 (62.86)	13 (37.14)
2–4 servings per day	204	157 (79.96)	47 (23.04)
>4 servings per day	21	17 (80.95)	4 (19.05)

* MET is the ratio of the rate of energy expended during an activity to the rate of energy expended at rest.

The distribution of sociodemographic and lifestyle factors in relation to education and income are presented in Table 2. Sex and age group were significantly different across different levels of education and income categories. Alcohol consumption and tobacco use were significantly different across education categories.

**Table 2:** Distribution of socioeconomic position in relation to age, sex, and lifestyle factors.

	**Education**				**Income**			
	**No formal education (*n* = 113)**	**Less than high school (*n* = 106)**	**High school or more (*n* = 41)**	***P*-value**	**Low income (*n* = 87)**	**Middle income (*n* = 87)**	**High income (*n* = 86)**	***P*-value**
**Sex**
Male	36 (31.9)	71 (67.0)	27 (65.8)	<0.001	51 (58.6)	41 (47.1)	34 (39.5)	0.041
Female	77 (68.1)	35 (33.0)	14 (34.2)		36 (41.4)	46 (52.9)	52 (60.5)	
**Age group (years)**
18–34	12 (10.6)	41 (38.7)	24 (58.5)	<0.001	15 (17.3)	23 (26.4)	39 (45.3)	<0.001
35–49	33 (29.2)	41 (38.7)	14 (34.2)		24 (27.6)	36 (41.9)	28 (32.7)	
50–65	35 (31.0)	17 (16.0)	3 (7.3)		21 (24.1)	19 (21.8)	15 (17.4)	
66 and above	33 (29.2)	7 (6.6)	0 (0.0)		27 (31.0)	9 (10.3)	4 (4.6)	
**Tobacco use**
Never	40 (35.4)	40 (37.8)	28 (68.3)	<0.001	33 (37.9)	31 (35.6)	44 (51.2)	0.219
Current	21 (18.6)	33 (31.1)	6 (14.6)		19 (21.8)	24 (27.6)	17 (19.8)	
Former	52 (46.0)	33 (31.1)	7 (17.1)		35 (40.2)	32 (36.8)	25 (29.1)	
**Alcohol intake**
Never	95 (84.1)	71 (66.9)	29 (70.7)	0.023	71 (81.6)	63 (72.4)	61 (70.9)	0.363
Low (<1 glass per week)	2 (1.8)	6 (5.7)	4 (9.8)		4 (4.6)	4 (4.6)	4 (4.7)	
Moderate (1–3 glass per week)	7 (6.2)	6 (5.7)	1 (2.4)		4 (4.6)	7 (8.1)	3 (3.5)	
High (>3 glass per week)	9 (7.9)	23 (21.7)	7 (17.1)		8 (9.2)	13 (14.9)	18 (20.9)	
**Physical activity**
MET^*^ <600 min/week	14 (12.4)	10 (9.4)	2 (4.9)	0.377	12 (13.8)	4 (4.6)	10 (11.6)	0.107
MET ≥600 min/week	99 (87.6)	96 (90.6)	39 (95.1)		75 (86.1)	83 (95.4)	76 (88.4)	
**Fruits and vegetables servings**
<2 servings per day	10 (8.8)	17 (16.0)	8 (19.5)	0.329	9(10.3)	11 (12.7)	15 (17.5)	0.567
2–4 servings per day	95 (84.1)	80 (75.5)	29 (70.7)		71 (81.6)	67 (77.0)	66 (76.7)	
>4 servings per day	8 (7.1)	9 (8.5)	4 (9.8)		7 (8.1)	9 (10.3)	5 (5.8)	

* MET is the ratio of the rate of energy expended during an activity to the rate of energy expended at rest.

In Table 3, the age-standardized prevalence of OWOB was higher among the high-income group, those who attained high school or had higher education, and the unemployed group. In the adjusted model, we observed that the prevalence of OWOB was 1.69 and 2.27 times greater in the high-income group and those with education of high school and above, respectively, compared to individuals in the low-income group and those with no formal education. Although the prevalence ratio was >1, there was uncertainty of the point estimates due to wide confidence interval. Furthermore, compared to the unemployed individuals, the farmers had significantly lower prevalence of OWOB (PR 0.5 and 95% CI 0.28–0.9). Furthermore, we found no evidence of effect modification on the outcome by age and sex. The *p*-value for interaction was not significant (results not shown).

**Table 3:** Multivariable modified Poisson regression analyses between socioeconomic positions and OWOB.

**Socioeconomic factors**	**Overweight/obesity *N* (%)**	**Age standardized^a^ prevalence (95%CI)**	**PR^b^^,^^c^ (95% CI)**	***P-*value**
**Income**				
Low	14 (16.09)	18% (10–26%)	Ref (1.0)	
Middle	19 (21.84)	21% (12–29%)	1.26 (0.66–2.42)	0.487
High	31 (36.05)	32% (22%-41%)	1.69 (0.92–3.14)	0.093
**Education**				
No formal education	21 (18.58)	19% (12–27%)	Ref (1.0)	
Less than high school	26 (24.53)	21% (13–28%)	1.51 (0.77–2.94)	0.233
High school and above	17 (41.46)	23% (10–35%)	2.27 (1.00–5.13)	0.049
**Employment status**				
Unemployed	17 (28.81)	42% (30–55%)	Ref (1.0)	
Farming	20 (15.63)	11% (6–17%)	0.50 (0.28–0.90)	0.020
Employed	27 (36.99)	26% (16–36%)	1.21 (0.68–2.15)	0.516

a Standardized to the WHO Standard Population,

b PR = prevalence ratio.

c Adjusted for age (continuous), gender (male, female), marital status (married, unmarried), and ethnicity (Brahmin, Chettri, Dalits, Other).

## Discussion

In this cross-sectional study, we assessed the effect of SES on OWOB among participants from rural Nepal. We found that OWOB was predominant among men, young adults, those married, moderate alcohol drinkers, non-tobacco users, and those less physically active. We observed a positive association between SES (education, income, employment status) and prevalence of OWOB, while the group of farmers had significantly lower prevalence of being overweight or obese compared to the unemployed group. Furthermore, we found no evidence of effect modification by sex and age.

In line with our findings, nationally representative surveys from Nepal (6, 34) and studies from other low-income countries (35–38) reported a positive SES and OWOB association. The prevalence of OWOB was reported to be higher among affluent and educated individuals in Nepal (6), Bangladesh (39) and South Asia (40).

On the contrary, studies from developed countries reported an inverse association between SES (education level and income level) and obesity (8, 15, 41). Nevertheless, a meta-analysis of prospective cohort studies from high-income countries suggested that the inverse association observed between SES and obesity was inconclusive after correcting for publication bias and reverse causality (that obese people were less likely to earn) (42). A systematic review from a developing country reported mixed results by gender, i.e., positive association for men and inverse for women (43).

The positive association observed between SES and obesity might be explained by the high-SES group having access to surplus food (44), and change in dietary pattern to consumption of high-fat and sugar-containing foods (8, 14, 45–47). Occupation is related to physical activity, and many jobs in Nepal are still labor-intensive (27); however, those with high SES in rural Nepal seem more likely to be OWOB, as they are often engaged in sedentary jobs (48). The high prevalence of obesity can also be explained by preference for large body sizes in some countries (8, 49–51), including Nepal (24) where large body size is considered a sign of economic prosperity, thus high SES may gain weight to maintain a status quo. On the other hand, the lower prevalence of obesity among those with low SES might be explained by poor availability of nutritious food (44) and engagement in high energy-expending jobs (48, 52).

Furthermore, the inconsistent results observed between the studies might also be due to different categorizations of variables such as obesity, income and education, heterogeneity of the study population, variables included in the model, and, more importantly, the different economic development stages of the countries (43). As a country's economy progresses, SES and obesity associations might also tend to be reversed (15). Moreover, studies from high- and middle-income countries achieving economic prosperity have shown the reversal of obesity gradient with increase in income occurring more swiftly (43). The difference in obesity and SES association in high- and low-income countries is determined by lifestyle choices; high-SES individuals in LMICs consume high-calorie foods and avoid physically demanding tasks while high-SES individuals in high-income countries tend to eat a healthy diet and regularly exercise (53). Nepal has achieved a moderate reduction in poverty with a steady increase in gross domestic product (35). Evidence suggests obesity is rising in low-resource settings including Nepal, with a higher increase reported among the rural population (18, 54). Therefore, Nepal needs to understand that obesity is no longer confined to affluent populations in urban areas.

The government of Nepal monitors obesity trends through routine surveys and tackles it through broader NCD policies and programming (55). However, unclear implementation mechanisms and being under resourcing of these policies hamper effective implementation (56). Furthermore, the association of obesity with adverse events such as stroke, cardiovascular events, and diabetes (57, 58) makes it urgent to address modifiable risk factors by launching an obesity prevention and management program in rural Nepal where primary healthcare facilities are not well-equipped and are in a tattered state.

Our study has a few strengths. First, a validated questionnaire was used for the data collection. Second, we measured weight and height instead of relying on self-reported measures for computing BMI.

Our study also has some limitations. First, our analysis was based on a relatively small sample size with reasonable statistical power limiting our ability to perform further sub-group analyses. Second, due to the cross-sectional nature of the study design, we cannot rule out the possibility of reverse causality. Third, the self-reported questionnaire on physical activity, alcohol use, and income data might have introduced recall bias (59). Lastly, we cannot rule out the possibility of residual confounding because of some unmeasured and incorrectly specified adjusted confounders.

## Conclusion

Overall, the findings from this study suggest that high-SES individuals had higher prevalence of OWOB. However, the results were based on participants who visited one health center in rural Nepal, limiting its generalizability even within regional Nepal.

## Recommendation

We recommend studies to understand how the SES and obesity relationship changes with socioeconomic development in Nepal. Similarly, larger studies are required to replicate our findings, preferably a large prospective cohort study from rural Nepal to demonstrate the SES and OWOB association among different population groups, and needed for timely identification of high-risk groups that will allow for efficient use of scarce health resources to develop effective and personalized interventions to prevent obesity in rural Nepal.

## Supplementary description

- The show cards shown to the respondents during data collection were same as the one used in the Non-Communicable Diseases Risk Factors: STEPS Survey Nepal 2013
  ° to identify the type of tobacco the respondents used
  ° to determine the amount of alcohol the respondents consumed
  ° to identify the type of fruits the respondents ate
  ° to identify the type of physical activity the respondents were engaged in.

## Data availability statement

The raw data supporting the conclusions of this article will be made available by the authors upon receiving request and approval to share from the ethics committee.

## Ethics statement

Ethical approval from the Regional Ethical Committee in Central Norway and Institutional Review Committee of Kathmandu University School of Medical Sciences Nepal was obtained. Informed consent was obtained before the start of data collection. The enumerators were trained in ethical consideration of human subject research to minimize breach in confidentiality. The data were de-identified for analysis. The identifiers were stored for 5 years in a locked cabinet.

## Author contributions

SB performed the analysis and drafted the manuscript. RN conceived the study, collected the data, and contributed to the draft. ASh and BK provided input during study design and on the drafting manuscript. ASe provided suggestions on data analysis and presentation, edited the draft of the manuscript, and approved the final version of the manuscript. All authors contributed to the article and approved the submitted version.

## Conflict of interest

The authors declare that the research was conducted in the absence of any commercial or financial relationships that could be construed as a potential conflict of interest.

## Publisher's note

All claims expressed in this article are solely those of the authors and do not necessarily represent those of their affiliated organizations, or those of the publisher, the editors and the reviewers. Any product that may be evaluated in this article, or claim that may be made by its manufacturer, is not guaranteed or endorsed by the publisher.
