# Supplementary material for: Association between socioeconomic positions and overweight/obesity in rural Nepal
Source: Front Nutr. 2022 Sep 9;9:952665. doi: 10.3389/fnut.2022.952665 (PMC9501994; doi:10.3389/fnut.2022.952665)

Supplementary Figures

Show cards used during Data collection

Source: Non-Communicable Diseases Risk Factors: STEPS Survey Nepal 2013

Supplementary Figure 1:Tobacco Products

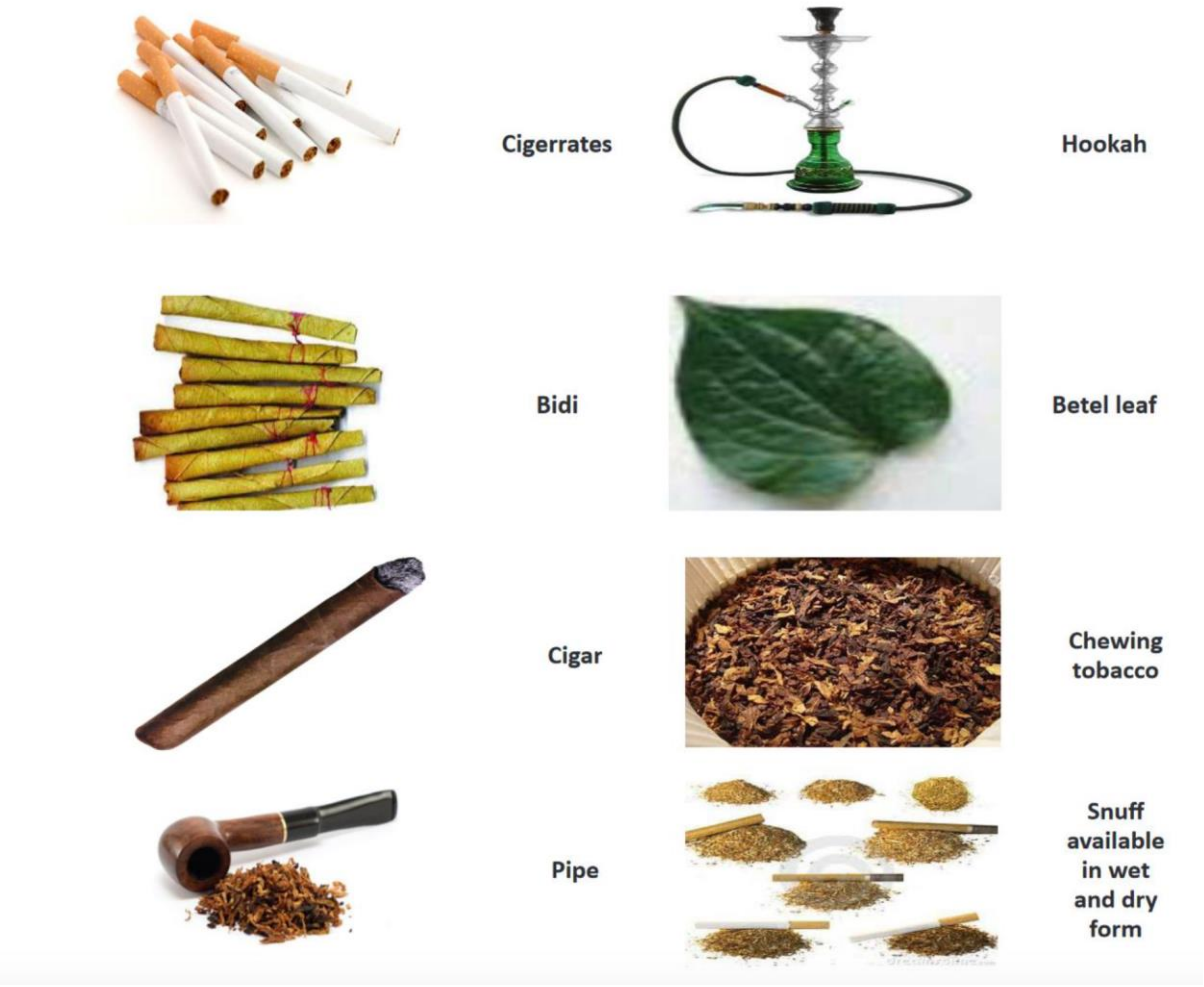

Supplementary Figure 2: Varieties of Glasses Used in Alcohol Consumption

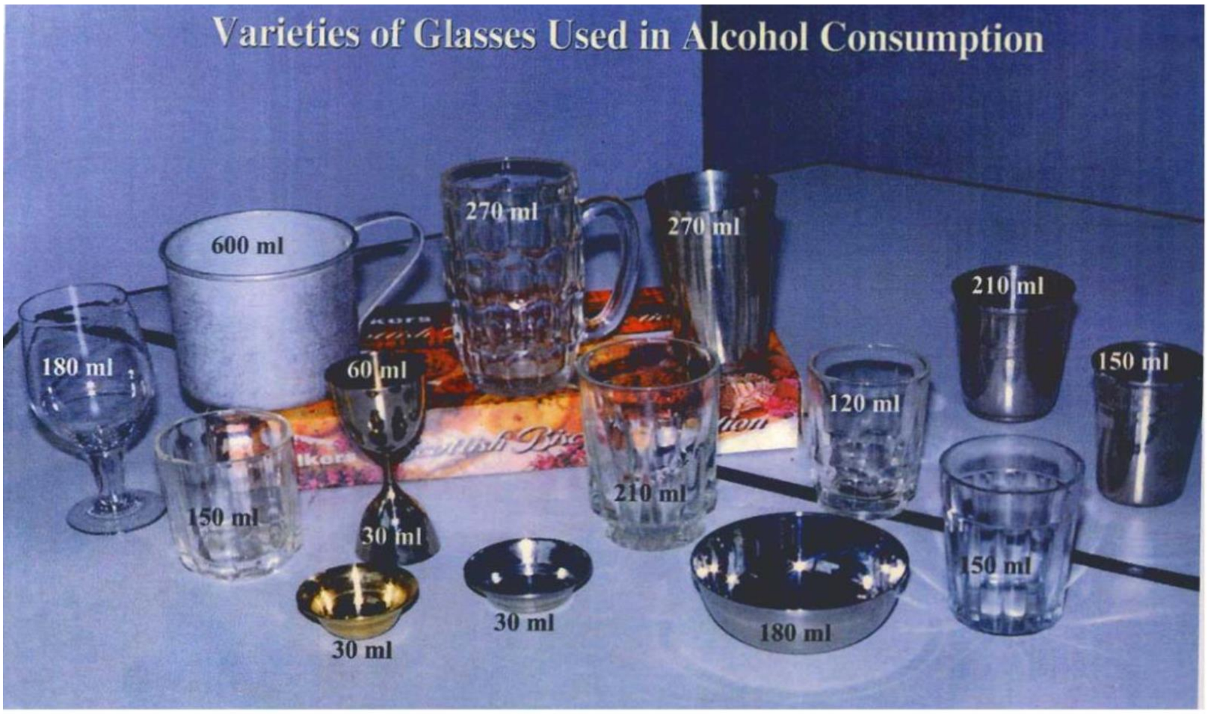

Supplementary Figure 3: Diet

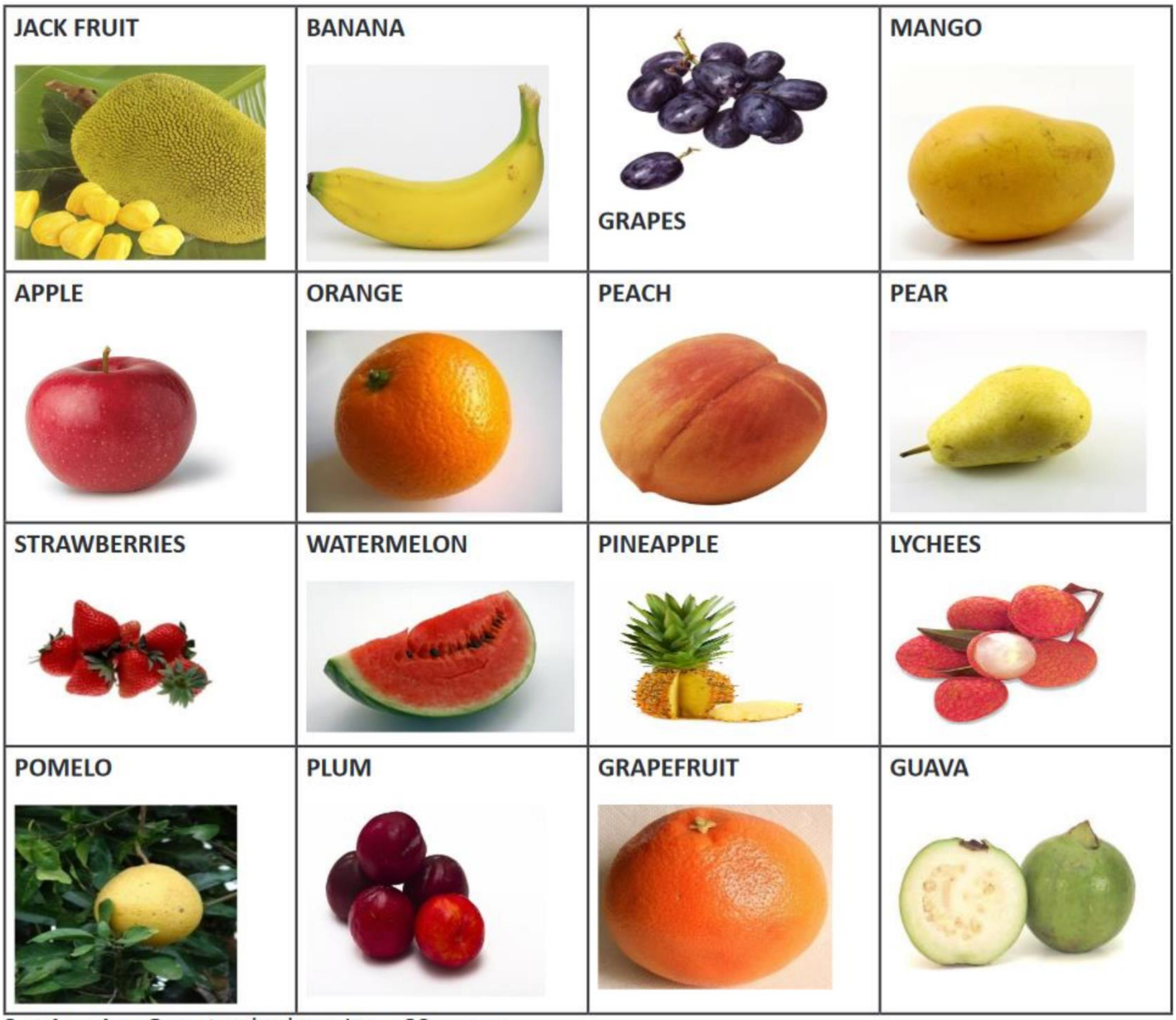

Serving size: One standard serving = 80 grams

Supplementary Figure 4: Typical Physical activities

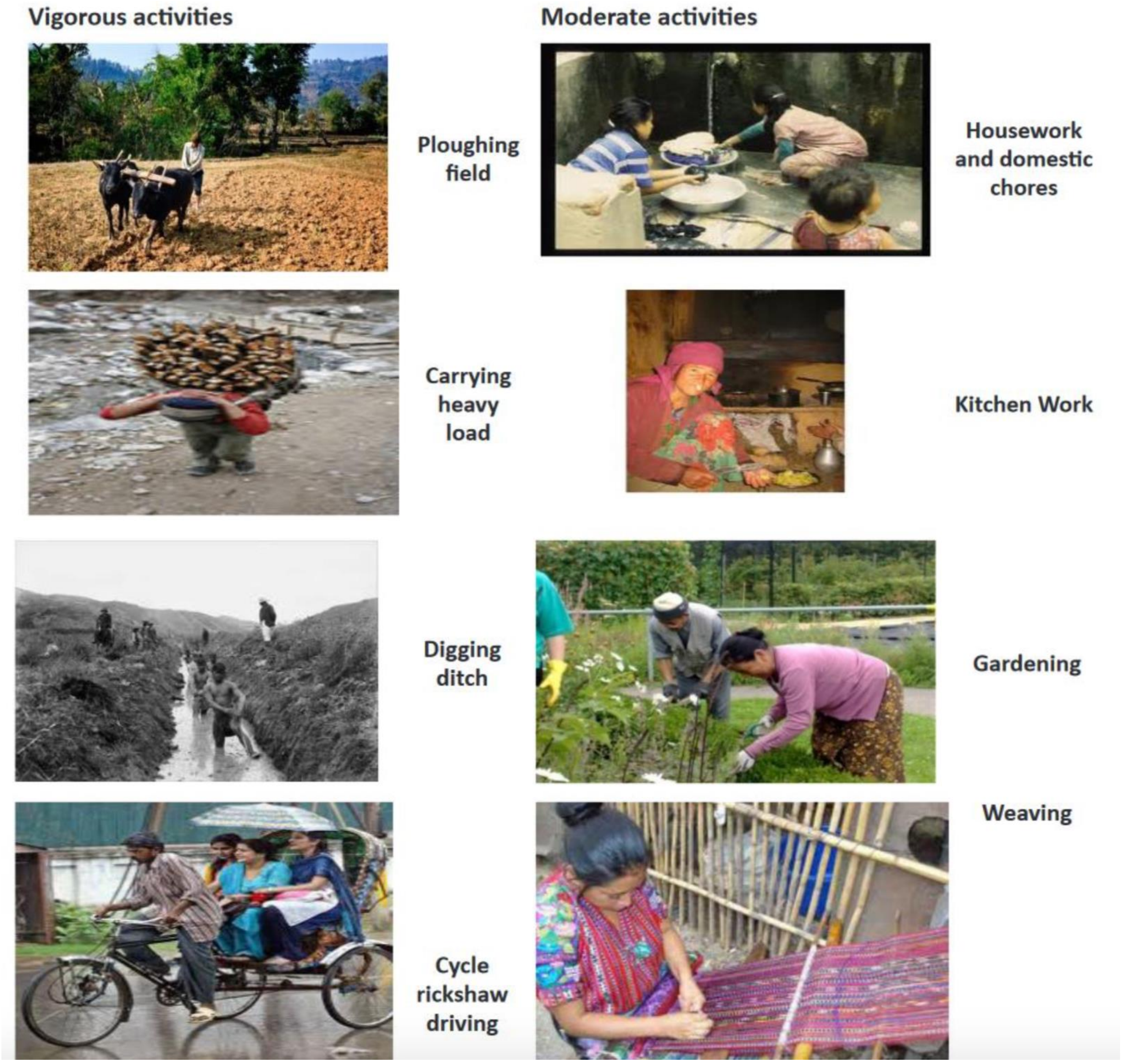

Supplement: Supplementary file 1 [file Data_Sheet_1.PDF]
